# Estimation of potato above-ground biomass based on unmanned aerial vehicle red-green-blue images with different texture features and crop height

**DOI:** 10.3389/fpls.2022.938216

**Published:** 2022-08-25

**Authors:** Yang Liu, Haikuan Feng, Jibo Yue, Xiuliang Jin, Zhenhai Li, Guijun Yang

**Affiliations:** ^1^Key Laboratory of Quantitative Remote Sensing in Agriculture of Ministry of Agriculture and Rural Affairs, Information Technology Research Center, Beijing Academy of Agriculture and Forestry Sciences, Beijing, China; ^2^Key Laboratory of Smart Agriculture System, Ministry of Education, China Agricultural University, Beijing, China; ^3^Key Laboratory of Agricultural Information Acquisition Technology, Ministry of Agriculture and Rural Affairs, China Agricultural University, Beijing, China; ^4^College of Agriculture, Nanjing Agricultural University, Nanjing, China; ^5^College of Information and Management Science, Henan Agricultural University, Zhengzhou, China; ^6^Institute of Crop Sciences, Chinese Academy of Agricultural Sciences/Key Laboratory of Crop Physiology and Ecology, Ministry of Agriculture, Beijing, China

**Keywords:** UAV, RGB images, GLCM-/Gabor-based textures, crop height, above-ground biomass

## Abstract

Obtaining crop above-ground biomass (AGB) information quickly and accurately is beneficial to farmland production management and the optimization of planting patterns. Many studies have confirmed that, due to canopy spectral saturation, AGB is underestimated in the multi-growth period of crops when using only optical vegetation indices. To solve this problem, this study obtains textures and crop height directly from ultrahigh-ground-resolution (GDS) red-green-blue (RGB) images to estimate the potato AGB in three key growth periods. Textures include a grayscale co-occurrence matrix texture (GLCM) and a Gabor wavelet texture. GLCM-based textures were extracted from seven-GDS (1, 5, 10, 30, 40, 50, and 60 cm) RGB images. Gabor-based textures were obtained from magnitude images on five scales (scales 1–5, labeled S1–S5, respectively). Potato crop height was extracted based on the generated crop height model. Finally, to estimate potato AGB, we used (i) GLCM-based textures from different GDS and their combinations, (ii) Gabor-based textures from different scales and their combinations, (iii) all GLCM-based textures combined with crop height, (iv) all Gabor-based textures combined with crop height, and (v) two types of textures combined with crop height by least-squares support vector machine (LSSVM), extreme learning machine, and partial least squares regression techniques. The results show that (i) potato crop height and AGB first increase and then decrease over the growth period; (ii) GDS and scales mainly affect the correlation between GLCM- and Gabor-based textures and AGB; (iii) to estimate AGB, GLCM-based textures of GDS1 and GDS30 work best when the GDS is between 1 and 5 cm and 10 and 60 cm, respectively (however, estimating potato AGB based on Gabor-based textures gradually deteriorates as the Gabor convolution kernel scale increases); (iv) the AGB estimation based on a single-type texture is not as good as estimates based on multi-resolution GLCM-based and multiscale Gabor-based textures (with the latter being the best); (v) different forms of textures combined with crop height using the LSSVM technique improved by 22.97, 14.63, 9.74, and 8.18% (normalized root mean square error) compared with using only all GLCM-based textures, all Gabor-based textures, the former combined with crop height, and the latter combined with crop height, respectively. Therefore, different forms of texture features obtained from RGB images acquired from unmanned aerial vehicles and combined with crop height improve the accuracy of potato AGB estimates under high coverage.

## Introduction

The above-ground biomass (AGB) of crops is the total dry organic mass of the above-ground vegetative organs per unit area at a particular time (Zhou et al., 2018). AGB is an essential phenotypic parameter for evaluating crop growth and predicting yield (Zhou et al., 2019). In addition, crop AGB information is a vital decision-making indicator for simulating nitrogen concentration dilution curves, which play an essential role in determining the nitrogen nutrition status and guiding fertilization management (Huang et al., 2015). Traditionally, manual measurement of AGB requires destructive sampling and weighing, which is a highly subjective, time-consuming, and labor-intensive manner to estimate from point to area (Liu et al., 2021a). At the same time, the constraints of sampling points and the variability of the field environment make this inefficient method to acquire AGB unsuited for large-scale real-time monitoring of crop growth (Castaldi et al., 2015). Therefore, new technologies are needed to quickly and accurately estimate the AGB of large-scale crops to provide scientific guidance for improving field management and increasing yield.

Remote-sensing technology is currently the most effective non-contact method for estimating crop AGB over large scales (David et al., 2020). To estimate the AGB of large-scale crops, satellite remote-sensing data (e.g., Sentinel-2, Landsat 8-OLI, and Worldview-2) is the best choice because of its advantages of wide coverage and free access by users (Dong et al., 2020; He et al., 2021). However, the satellite remote-sensing technology is difficult to fully exploit because of various factors such as satellite revisit cycle, atmospheric conditions, and spatial resolution, limiting the rapid development of precision agriculture (Li et al., 2020a). Although few restrictions encumber ground remote sensing based on backpacks or vehicle-mounted equipment, such an approach is unsuited for large-scale AGB monitoring because of the limitations of remote-sensing platforms (Ryu et al., 2020). Fortunately, remote sensing from unmanned aerial vehicles (UAVs) now fills the technology gap formed by and inadequacies of satellite and ground remote sensing. Given its simple operation, convenience, flexibility, efficient data acquisition, and high temporal and spatial resolution of the images obtained by UAV remote sensing, this approach has created a new paradigm for the quantitative estimation of crop AGB (Duan et al., 2021).

Vegetation indices (VIs) such as the normalized difference vegetation index, the renormalized difference vegetation index, and the ratio vegetation index obtained from broadband multispectral data from the visible to the near-infrared have been used with significant success to estimate crops AGB (Chen et al., 2009; Meng et al., 2013). At present, research into estimating AGB is mainly divided into two categories: (i) research based on physical models and (ii) research based on empirical regression models (Yang et al., 2019). Physical models (e.g., the PROSPECT and PROSAIL models) have robust mechanisms and applicability, but many parameters required by these models are difficult to obtain, which limits their use for estimating crop AGB (Wan et al., 2021; Yang et al., 2021a). In empirical models, different regression techniques are used to relate feature parameters to AGB (Meng et al., 2017; Xu et al., 2018). These regression techniques fall into two categories (Yue et al., 2018a): traditional regression techniques [e.g., multiple stepwise regression, partial least squares regression (PLSR), and principal component analysis] and machine-learning techniques [e.g., random forest, artificial neural networks, and extreme learning machine (ELM)]. Spectral VIs combined with various regression techniques to estimate crop AGB offer the advantages that (i) VIs and AGB are strongly correlated over the reproductive growth period of crops, and (ii) the model structure is simple, which facilitates applications in AGB estimation (Bao et al., 2020; Kumar et al., 2021).

However, numerous studies have challenged the wisdom of using VIs to estimate the crop AGB in the multi-growth periods. The main arguments brought to bear are that (i) VIs saturate easily under high crop coverage, and (ii) VIs lose their sensitivity to AGB in the multi-growth period, making it challenging to use VIs to estimate crop AGB in the multi-growth period (Ma et al., 2019; Zhang et al., 2020). At present, the four primary techniques used to enhance the accuracy of AGB estimates are (i) the synthetic aperture radar technique (Montesano et al., 2013, 2014), (ii) the laser intensity direction and ranging (LiDAR) technique (Cao et al., 2018; Hu et al., 2020), (iii) the narrowband hyperspectral technique (Filippi et al., 2014; Tao et al., 2020), and (iv) the crop-height model (CHM; Roth and Streit, 2018; Zhu et al., 2019a; Issa et al., 2020).

Given the use of long-wavelength electromagnetic radiation, synthetic aperture radar remote-sensing techniques can penetrate the crop canopy and are not affected by weather conditions, which means that they overcome the problem of premature saturation of AGB estimates by optical remote-sensing VIs and are thus highly suitable for long-term monitoring of AGB for areas with high crop coverage (Banda and Tebaldini, 2020). Previous studies have confirmed that using the backscattering coefficient of synthetic aperture radar remote sensing produces highly accurate estimates of crop AGB for wheat (Han et al., 2019), maize (Hosseini et al., 2019), and rice (Yang et al., 2016).

LiDAR remote-sensing techniques actively transmit electromagnetic wave pulses with a specific penetration ability to interact with ground objects. By statistically calculating the height or quantity of LiDAR echoes, different variables characterizing crop canopy structure (such as volume and coverage) can be obtained, which is very useful for estimating crop AGB (Malambo et al., 2018). For example, Walter et al. (2019) demonstrated that volume and height data acquired by LiDAR correlate strongly with wheat AGB. Wang et al. (2017) showed that VIs combined with metrics obtained through LiDAR improve estimates of maize AGB. Furthermore, because crop height is a good metric of crop growth, the canopy height determined by LiDAR to estimate crop AGB is a very promising method to resolve spectral saturation (Salum et al., 2020). At present, UAV-LiDAR is widely used, mainly because it is convenient, data collection is fast, it provides a digital elevation model and digital surface model of the field, and it promotes the production of a CHM, which provides a new avenue for estimating crop AGB (Zhu et al., 2019b).

Narrowband hyperspectral techniques have the capacity to continuously acquire crop canopy spectral reflectance data with high spectral resolution, which means that mining the hidden information in the spectrum is helpful for AGB estimation. Therefore, crop AGB estimation can be enhanced by using spectral differential analysis (Gnyp et al., 2014), band-depth analysis (Fu et al., 2014), continuous wavelet analysis (Yao et al., 2018), and red-edge-region analysis (Marshall and Thenkabail, 2015). For example, Yue et al. (2021) showed that the accuracy of estimates of winter wheat AGB during the multi-growth period could be improved by using wavelet coefficients and multiple stepwise regression methods. Yang et al. (2021b) proposed that, during the multi-growth period of crops, the red-edge region leads to more accurate AGB estimates than the use of traditional VIs.

Although the above-mentioned remote-sensing techniques accurately estimate AGB and allow for real-time monitoring of crop growth, obtaining the data incurs high cost, and data processing is complex, which prevents this approach from gaining wide acceptance in the private sector (Poley and McDermid, 2020). In contrast, UAV digital remote-sensing systems are more acceptable because of their low price, simple data structure, and convenient data processing (Guo et al., 2021; Wang et al., 2021). More importantly, UAV-based RGB images may be spliced together to obtain digital surface models (from which a CHM can be developed) and ultrahigh-ground-resolution (GDS) digital orthophoto images (from which crop canopy spectra can be extracted), which provides more avenues to accurately estimate crop parameters.

Previous reports have confirmed that the CHM generated from UAV-based RGB images can be used for AGB estimation (Yue et al., 2018b; Lu et al., 2019). However, estimating AGB in multiple growth periods based only on crop height is not reliable because the change in crop height (such as wheat, maize, and rice) is not apparent in the late growth period, whereas AGB continues to increase, making it unfeasible to estimate AGB in the whole growth period based only on crop height (Fu et al., 2021). Niu et al. (2019) reported that combining crop canopy height and VIs is more accurate for estimating maize AGB than the use of either crop height or VIs alone. Similarly, VIs calculated from RGB-based images also suffer from spectral saturation under high crop coverage, resulting in inaccurate AGB estimates (Christelle and Emmanuel, 2020). Thus, considering the limitations of VIs and crop height to estimate AGB, researchers have begun to mine image features from ultrahigh-GDS RGB images to enhance the accuracy of AGB estimation models.

UAV-based ultrahigh-GDS RGB images are helpful not only for estimating AGB (Batistoti et al., 2019) but also for estimating chlorophyll (Liu et al., 2021b), nitrogen content (Li et al., 2015), leaf area index (Yue et al., 2018a), and crop yield (Zeng et al., 2021). These studies show that ultrahigh-GDS RGB images are rich in crop canopy surface information for monitoring growth. Therefore, canopy texture features can be extracted from ultrahigh-GDS UAV-based RGB images for estimating AGB (Mao et al., 2021). For example, Yue et al. (2019) confirmed that textures based on the gray level co-occurrence matrix (GLCM) and extracted from UAV-based RGB images with various GDS produce more accurate winter wheat AGB estimates than the traditional narrow and wideband VIs. Zhu et al. (2021) also reported that GLCM-based textures from UAV-based RGB images can be used to estimate maize AGB.

A literature review shows that most studies only extract single-scale GLCM-based texture features from specific GDS images to estimate crop AGB. However, the Gabor-based transformation features provide information that can be used to describe image textures and have been fully applied in the field of image processing, despite receiving little attention in crop phenotyping research (Shen and Bai, 2006; Fu et al., 2021). The crop canopy structure and size are known to vary with the growth period, which makes it difficult to use single-scale texture features to reflect differences in canopy structure. If the multiscale texture features can be extracted, the morphology of the crop canopy structure could be described to maximum extent, allowing more accurate estimates of crop AGB over multiple growth periods.

Furthermore, the texture features extracted from ultrahigh-GDS UAV-based RGB images can represent the high-frequency information of crop canopy photos, which provides new information (e.g., lush vegetation) about the crop canopy. If this information can be combined with the vertical height of the crop canopy, it could be used to accurately estimate AGB. On the one hand, different texture techniques offer different potentials for extracting high-frequency information. On the other hand, the high-frequency information contained in images of different GDS will vary. After a careful literature review, no study has been found that proposes using different multiscale texture techniques to extract high-frequency information for estimating potato AGB.

Therefore, the present study investigates the performance of GLCM- and Gabor-based textures and various textures combined with crop canopy height to estimate potato AGB. GLCM-based textures were extracted from seven-GDS (1, 5, 10, 30, 40, 50, and 60 cm) RGB images. Gabor-based textures were obtained from magnitude images on five scales (scales 1–5, denoted S1–S5, respectively). We estimate the potato AGB by applying a least-squares support vector machine (LSSVM), an ELM, and PLSR using, respectively, (i) GLCM-based textures from different GDS RGB images and their combinations, (ii) Gabor-based textures from different scales and their combinations, and (iii) GLCM-based textures, Gabor-based textures, and their combinations integrated with crop height.

## Experiment and methods

### Experiments

The experiment was conducted at the National Precision Agriculture Research Demonstration Base (40°10’N, 116°26′E), Changping District, Beijing, China. Changping District has a typical warm temperate semi-humid continental monsoon climate, where the main crops are summer maize and winter wheat.

**Figure 1:**
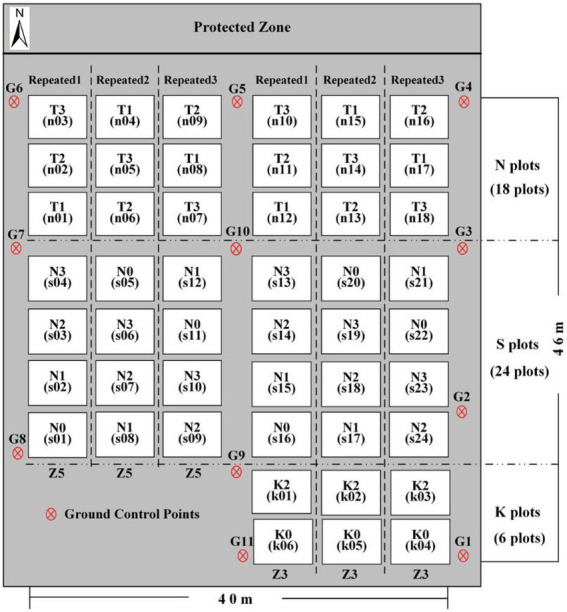
Potato planting plan at Changping District, 2019.

To increase the spatial growth difference of potato crops in the field, we use Zhongshu 5 (Z5) and Zhongshu 3 (Z3) early maturing potato varieties planted with different planting densities, nitrogen, and potassium fertilizers treatments. Forty-eight plots were planted, with each plot covering 32.5 m^2^ and with a row spacing of 0.6 m. Eighteen plots filled the density test area with three gradients [60,000 plants/hm^2^ (T1), 72,000 plants/hm^2^ (T2), and 84,000 plants/hm^2^ (T3)] and six repetitions. Twenty-four plots occupied the nitrogen test area with four gradients [0 kg/hm^2^ urea (N0), 244.65 kg/hm^2^ urea (N1), 489.15 kg/hm^2^ urea (N2, normal treatment, 15 kg pure N), and 733.50 kg/hm^2^ urea (N3)] and six repetitions. Six plots occupied the potassium fertilizer test area with two gradients [0 kg/hm^2^ potassium fertilizer (K0), 1941 kg/hm^2^ potassium fertilizer (K2)] and three repetitions. The specific test plan is shown in Figure 1. The planting method is mulching, and the field management includes weeding, soil cultivation, and watering. To accurately correct the terrain for subsequent RGB images, eleven ground control points (G1–G11) were uniformly deployed around the test plot and accurately positioned by using differential global positioning with millimeter accuracy.

### Collection and processing of crop height and AGB data

Ground crop height and AGB data were collected on 28 May 2019, 10 June 2019, and 20 June 2019, which corresponded to periods of potato tuber formation (P1), tuber growth (P2), and starch storage (P3), respectively. During each growth period, four plants representative of the overall growth level were selected from each plot. The vertical height of each plant was measured with a ruler, and the average value was taken as the measured crop height (in centimeters) for each plot.

To obtain the AGB data, three plants representative of the overall growth level were artificially selected from each plot. After artificial field sampling, put it into a white sealed bag and quickly took it back to the laboratory. The samples were washed with running water indoors. After the sample was naturally dried, the stems and leaves were cut into small pieces by using scissors. The separated samples were killed at 105°C, and dried at 80°C in a large bake oven until reaching constant mass. For each growth period, the plant density and dry mass of the stems and leaves of each plant sample as measured by a high-precision balance were used to calculate the potato AGB of each plot (in kg/hm^2^).

### Unmanned aerial vehicle RGB image acquisition and processing

After the ground collection work was completed, the DJI 4A series product produced by DJI Group, Ltd. was used to carry out UAV remote-sensing operations in the bare soil period (April 20, 2019) and the three critical growth periods of potatoes. The UAV system was equipped with a three-channel CMOS sensor, with 20 million effective pixels and a maximum pixel value of 4,000 × 3,000. To ensure the generation of RGB images with high GDS, the UAV’s flying height was manually set to 20 m, and the heading and side overlap were both 80%. By using its position and orientation system during flight, the UAV recorded the three-dimensional position of the sensor in real-time. The flights were conducted in clear, calm weather to reduce the variation of crop canopy reflection intensity caused by uneven illumination. The take-off position and flight path were essentially the same for each flight to accurately match the digital surface model (DSM) obtained in different growth periods.

Before extracting crop height and multiscale texture features from UAV RGB images, we used Agisoft PhotoScan Professional software to splice together digital images from the various periods. Next, the RGB images were topographically corrected based on the measured three-dimensional coordinates of G1–G11, and the correction error for each period was less than 2 cm. Finally, the DSM and digital orthophoto map of the test location were derived for each period. The specific image-processing flow followed that of Fu et al. (2021). A total of 48 regions of interest (ROIs) were delineated according to the boundary of each sample plot.

### Generation of crop height model

**Figure 2:**
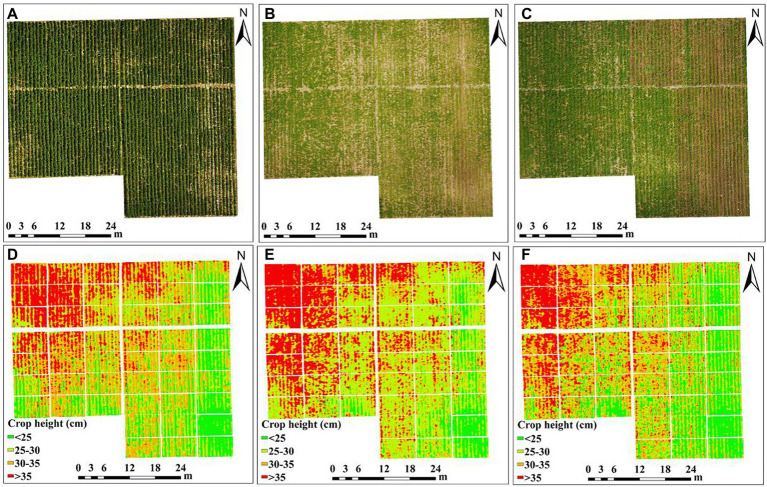
UAV RGB images and potato crop height of each plot. **(A,D)** P1, **(B,E)** P2, and **(C,F)** P3 periods.

Crop height represents the growth of groups and symbolizes the vertical structure of the crop canopy, which is closely related to AGB. Therefore, the accurate acquisition of crop-height information is important for monitoring crop growth and managing farmland production (Niu et al., 2019). First, in this study, the DSM of the three critical growth periods (P1–P3) and the bare soil period of potatoes were topographically corrected and established through high-density point clouds. The raster statistics tool of ArcGIS 10.2 software was then used to calculate the difference in DSM between potato critical growth periods and the bare soil period and obtain the crop height model of the corresponding growth period (Figures 2D–F). Finally, the average potato crop height of each plot was automatically extracted by using the ROI tool.

### Extraction of GLCM- and Gabor-based texture features

The GLCM is a matrix function involving pixel distance and angle. It reflects spatial variations in the gray distribution of the image by calculating the correlation between the gray levels of two points separated by a certain distance and in a specific direction in the image (Fu et al., 2021). The flying height of the UAV in this study was 20 m, and the GDS of the obtained images was about 0.85 cm. The original image was sampled to 1 cm by resampling. GDS5, GDS10, GDS20, GDS30, GDS40, GDS50, and GDS60 cm images were acquired based on the GDS1-cm image using the nearest-neighbor pixel method. In this study, GLCM-based texture features in four directions (0°, 45°, 90°, 135°) were extracted from each channel of UAV RGB images. Four moving windows (3 × 3, 5 × 5, 7 × 7, 9 × 9) were set in each direction for extracting diverse textures. We selected eight standard GLCM-based texture features, including the mean (Mea), variance (Var), homogeneity (Hom), contrast (Con), dissimilarity (Dis), entropy (Ent), second moment (Sec), and correlation (Cor) for analyzing the performance of estimating AGB. To simplify the description, the texture features were prefixed with R-, G-, or B- to denote the GLCM-based extracted textures for the three channels (e.g., R-Con denotes the contrast of the R band).

**Figure 3:**
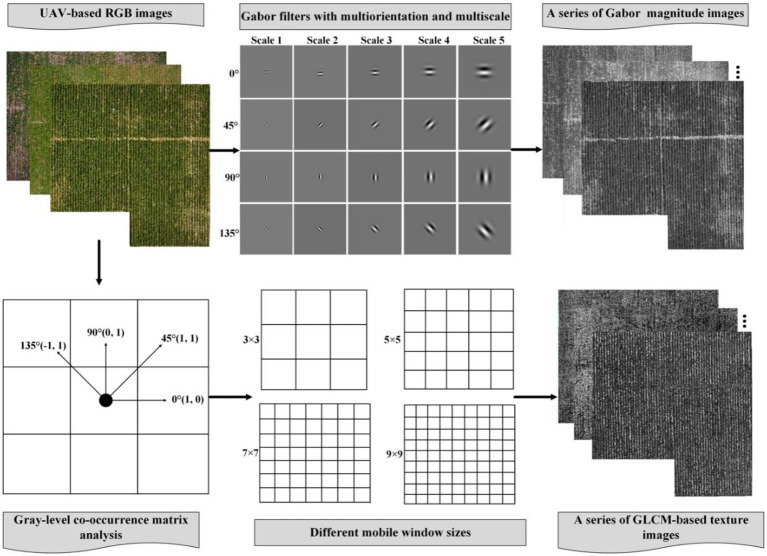
Multiform texture features extraction process from GLCM- and Gabor-based analysis.

The Gabor transform, also known as the windowed Fourier transform (Gaussian function as a window function), is a transformation from the time to the frequency domain and offers good characteristics for extracting local space and frequency domain information from the target (Shen and Bai, 2006). The Gabor filter is like the visual stimulus–response of simple cells in the human visual system. It is sensitive to the edge of the image, which can provide good direction-scale selection characteristics, and is insensitive to illumination changes, confronting changes in illumination with appropriate adaptation (Jones and Palmer, 1987; Fu et al., 2020). Therefore, the Gabor transform is often used to extract and analyze image texture features. In this study, four directions (0°, 45°, 90°, 135°) and five scales (S1–S5) were selected to generate a total of twenty filter banks. The other parameters were set to the same values as in Fu et al. (2021). For each ROI in the growth period of each potato, the RGB images were convolved with the Gabor filter banks to generate a total of sixty types of amplitude images. Based on these magnitude images, we obtained the same textures as GLCM-based textures. Therefore, 480 texture features were extracted from each plot. To simplify the description later, R-, G-, B- followed by the scale factors served to characterize the texture features of different bands and scales (e.g., R-S2-Ent denotes the entropy of scale 2 of the R-band). Figure 3 shows the specific process of extracting diverse GLCM- and Gabor-based textures.

### Technical route and regression analysis

**Figure 4:**
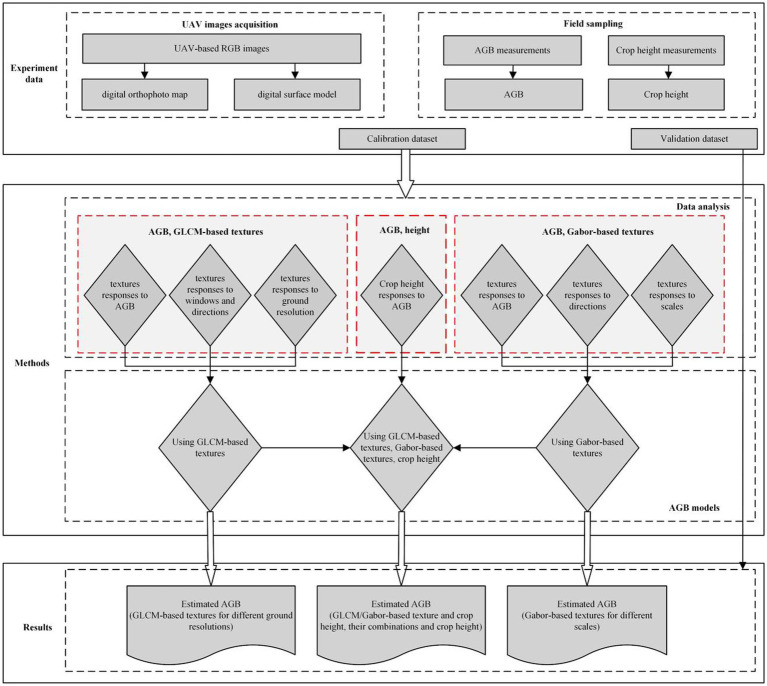
Technical route of the study.

To verify the hypothesis proposed herein, repeats 2 and 3 data (32 groups) collected in each potato growth period in 2019 served as the calibration set to estimate AGB, and repeat 1 data (16 groups) served as the validation set to verify the reliability and stability of the model. Figure 4 shows the technical scheme of this study. The coefficient of determination R (Zhou et al., 2019), root mean square error (RMSE), mean absolute error (MAE), and normalized root mean square error (NRMSE) were used to evaluate the estimation accuracy of different models.

## Results and analysis

### Crop-height response to potato AGB

**Figure 5:**
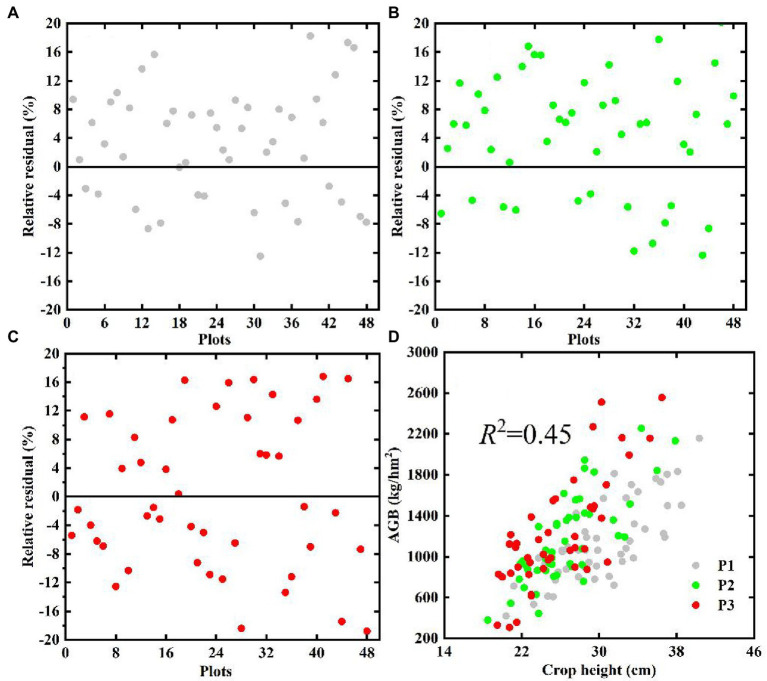
Comparison of the relative residuals (%) of the extracted crop height during the growth period of potato **(A)** P1, **(B)** P2, **(C)** P3, and **(D)** the relationship between AGB and estimated crop height based on UAV.

Figures 5A–C show the relative residuals of the extracted crop height based on UAV RGB images during the P1–P3 growth period of potatoes. The results show that the extracted crop height in each growth period is generally low, and that most of the relative residuals are less than 20% (RMSE <3 cm), which indicates that the crop height extracted through the crop height model (Figure 2) is reliable. The result in Figure 5D shows that crop height and AGB correlate positively in all key potato growth periods (*p* > 0.01), which means that crop height may contribute to the potato AGB estimates over multi-growth periods.

### Response of GLCM-based texture features to potato AGB

#### Response of GLCM-based textures from different windows and directions to potato AGB

**Figure 6:**
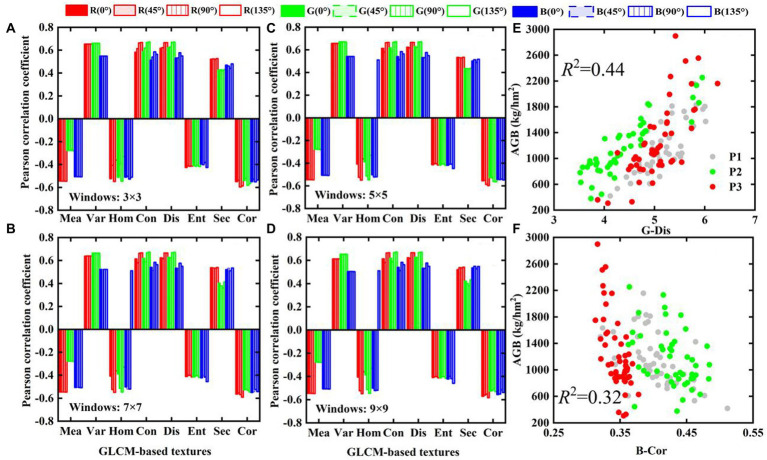
The relationship between GLCM-based textures and AGB in different directions and different windows: **(A)** 3 × 3, **(B)** 5 × 5, **(C)** 7 × 7, **(D)** 9 × 9, **(E,F)** scatter plots of G-Dis and B-Cor with AGB under 45° and 5 × 5 windows, respectively.

Taking the RGB images of GDS1 as an example, Figures 6A–D examine the Pearson correlation coefficients between GLCM-based textures and potato AGB in different windows and directions. The results show that the correlation of GLCM-based textures with potato AGB is basically independent of direction and window size. To reduce the dimensionality of the data, we use GLCM-based textures with 45° orientation and a 5 × 5 window for potato AGB estimation. The results shown in Figures 6E,F indicate that GLCM-based texture correlates weakly with potato AGB in three single growth periods (*p* < 0.05), and that, when considering the multiple growth periods of crops, GLCM-based textures correlate positively (e.g., G-dis) or negatively (e.g., B-cor) with potato AGB (*p* < 0.01), which indicates that the use of GLCM-based textures may also improve the accuracy of AGB estimates of potatoes in multiple growth periods.

#### Response of GLCM-based textures of different ground resolution to potato AGB

**Figure 7:**
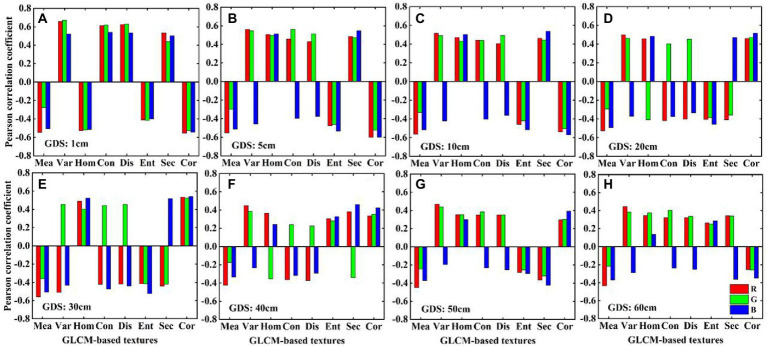
The relationship between different ground resolution (GDS) GLCM-based textures and AGB. **(A)** 1, **(B)** 5, **(C)** 10, **(D)** 20, **(E)** 30, **(F)** 40, **(G)** 50, **(H)** 60  cm image textures.

The results shown in Figure 7 indicate that the Pearson correlation coefficients of GLCM-based textures with different GDS and potato AGB differ significantly. GLCM-based textures of GDS1 correlate strongly with AGB (*p* < 0.01). The GLCM-based textures of GDS5 and GDS10 correlate more weakly with AGB and the correlation coefficient has the opposite sign. The GLCM-based textures of GDS20 and GDS30; GDS40, GDS50, and GDS60 approximately correlate with AGB. These results show that the image GDS affects the relationship between GLCM-based textures and AGB. Therefore, we must evaluate the accuracy of GLCM-based textures at different resolutions to estimate potato AGB.

### Response of Gabor-based texture features from different directions and scales to potato AGB

**Figure 8:**
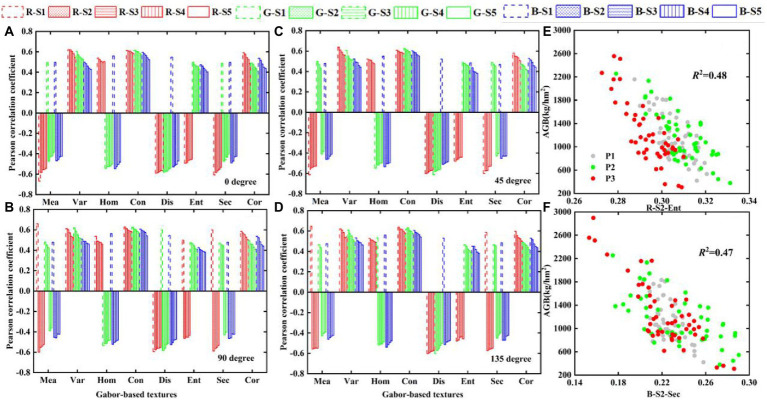
The relationship between Gabor-based textures and AGB in different directions and different scales: **(A)** 0-degree, **(B)** 45-degree, **(C)** 90-degree, **(D)** 135-degree, **(E,F)** scatter plots of R-S2-Ent and B-S2-Sec with AGB, respectively.

Figures 8A–D show the Pearson correlation coefficients of Gabor-based textures (GDS1) and potato AGB at different orientations and different scales. The size and sign of the correlation coefficient show that the correlation between Gabor-based textures and potato AGB is significantly more affected by scale than by direction, which differs completely from the result for GLCM-based textures. Therefore, we use Gabor-based textures with different scales in 45° orientation to estimate potato AGB. The results shown in Figures 8E,F indicate an excellent linear relationship between Gabor-based textures and potato AGB in single- or multi-growth periods under multiple scales (*p* < 0.01). Compared with the results in Figures 7E,F, when the AGB exceeds 1,000 kg/hm^2^, the Gabor-based textures remain sensitive to AGB (*p* < 0.01), which indicates that the multiscale Gabor-based textures are more promising for AGB estimation than multi-resolution GLCM-based textures.

### Using GLCM-based texture features to estimate potato AGB

As shown in Figure 7, five representative ground resolution images were selected to extract GLCM-based textures to estimate potato AGB. The selected ground resolution and GLCM-based textures (using the “findCorrelation” function in the Caret package of the R language, with a cutoff of 0.99) appear in Table 1.

**Table 1:** Selected GLCM-based textures to estimate potato AGB.

Textures	Ground resolution (cm)	Parameters
GLCM-based textures	GDS1	R-Mea, R-Var, R-Con, R-Dis, R-Cor, G-Var, G-Con, G-Dis, B-Con, B-Cor
	GDS5	R-Mea, R-Cor, G-Con, G-Dis, G-Cor, B-Mea, B-Hom, B-Ent, B-Sec, B-Cor
	GDS10	R-Mea, R-Hom, G-Var, B-Mea, B-Var, B-Hom, B-Con, B-Ent, B-Sec
	GDS30	R-Mea, B-Mea, B-Var, B-Hom, B-Con, B-Dis,
	GDS60	R-Mea, G-Var, G-Con, G-Dis, B-Mea, B-Var, B-Dis

**Figure 9:**
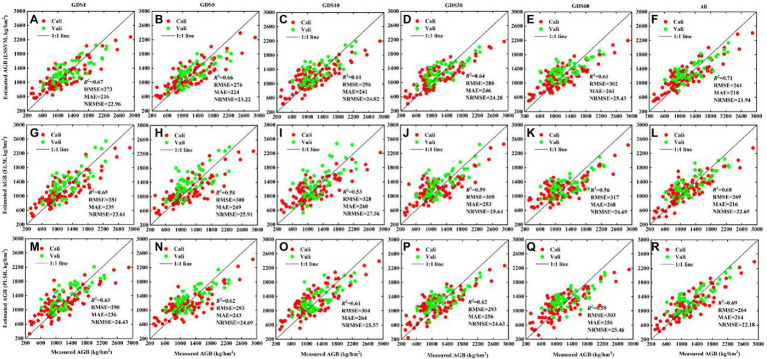
The fitted scatter plot of measuring and estimating potato AGB (kg/hm^2^) using GLCM-based textures from GDS1, GDS5, GDS10, GDS30, GDS60 and all, respectively. **(A–F)** LSSVM; **(G–L)** ELM; **(M–R)** PLSR. “All” represent texture combinations of different ground resolutions. Cali and Vali represent calibration (repeat 2 and 3) and validation data sets (repeat 1), respectively. The estimation results of Cali and Vali are shown in Supplementary Table A1.

Figure 9 shows potato AGB estimates using the LSSVM, ELM, and PLSR techniques based on image textures of GDS1, GDS5, GDS10, GDS30, GDS60, and their combinations. These results show that multi-resolution GLCM-based textures provide better estimates (*R*^2^ = 0.68–0.71, RMSE = 261–269 kg/hm^2^, MAE = 214–218 kg/hm^2^, NRMSE = 21.94–22.65%) than others with single ground resolution textures. In addition, for GDS1 to GDS5, the GLCM-based textures of GDS1 provide the best AGB estimates (*R*^2^ = 0.63–0.67, RMSE = 273–290 kg/hm^2^, MAE = 216–236 kg/hm^2^, NRMSE = 22.96–24.43%). Between GDS10 and GDS60, the GLCM-based textures of GDS30 provide the best AGB estimates (*R*^2^ = 0.59–0.64, RMSE = 288–305 kg/hm^2^, MAE = 246–256 kg/hm^2^, NRMSE = 24.28–25.64%).

### Using Gabor-based texture features to estimate potato AGB

The multiscale Gabor-based textures used in this study to estimate potato AGB are listed in Table 2 (the selection rules are given in Table 1). To compare the effect of single and multiscale Gabor-based textures for potato AGB estimation, we also use the LSSVM, ELM, and PLSR techniques to build AGB estimation models.

**Table 2:** Selected Gabor-based textures to estimate potato AGB.

Textures	Scales	Parameters
Gabor-based textures	S1	R-S1-Var, R-S1-Ent, R-S1-Cor, G-S1-Var, B-S1-Var, B-S1-Cor
	S2	R-S2-Hom, R-S2-Con, R-S2-Dis, R-S2-Ent, R-S2-Sec, R-S2-Cor, G-S2-Var, B-S2-Cor
	S3	R-S3-Mea, R-S3-Hom, R-S3-Con, R-S3-Dis, R-S3-Sec, B-S3-Var, B-S3-Cor
	S4	R-S4-Hom, R-S4-Con, R-S4-Dis, B-S4-Hom, B-S4-Var
	S5	R-S5-Mea, R-S5-Hom, R-S5-Con, R-S5-Dis, R-S5-Sec, B-S5-Var

**Figure 10:**
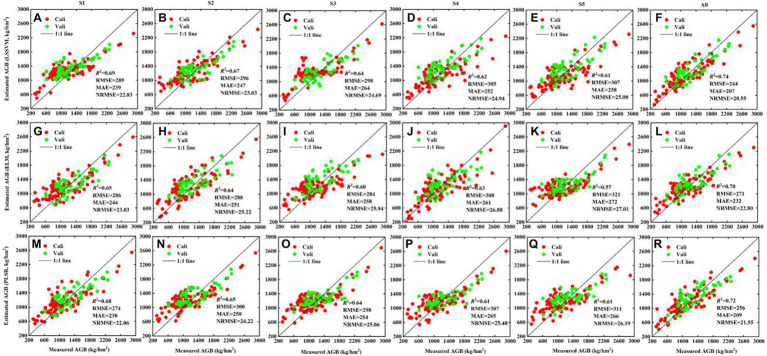
The fitted scatter plot of measuring and estimating potato AGB (kg/hm^2^) using Gabor-based textures from S1, S5, S3, S4, S5 and all, respectively. **(A–F)** LSSVM; **(G–L)** ELM; **(M–R)** PLSR. “All” represent texture combinations of different scales. The estimation results of Cali and Vali are shown in Supplementary Table A2.

The results in Figure 10 show that (i) the use of multiscale Gabor-based textures produces more accurate AGB estimates than the use of GLCM-based textures with different GDS (*R*^2^ = 0.70–0.74, RMSE = 244–271 kg/hm^2^, MAE = 207–232 kg/hm^2^, NRMSE = 20.55–22.80%). (ii) For different scales, the AGB estimation results gradually became less accurate with increasing scale. (iii) Finally, when the AGB exceeds 1,000 kg/hm^2^, the multiscale Gabor-based textures (Figures 10F,L,R) overestimates the AGB more than does the GLCM-based textures of different GDS. These results confirm that the multiscale Gabor-based textures produce more accurate estimates of potato AGB than do multi-resolution GLCM-based textures.

### Using texture features and crop height to estimate potato AGB

**Figure 11:**
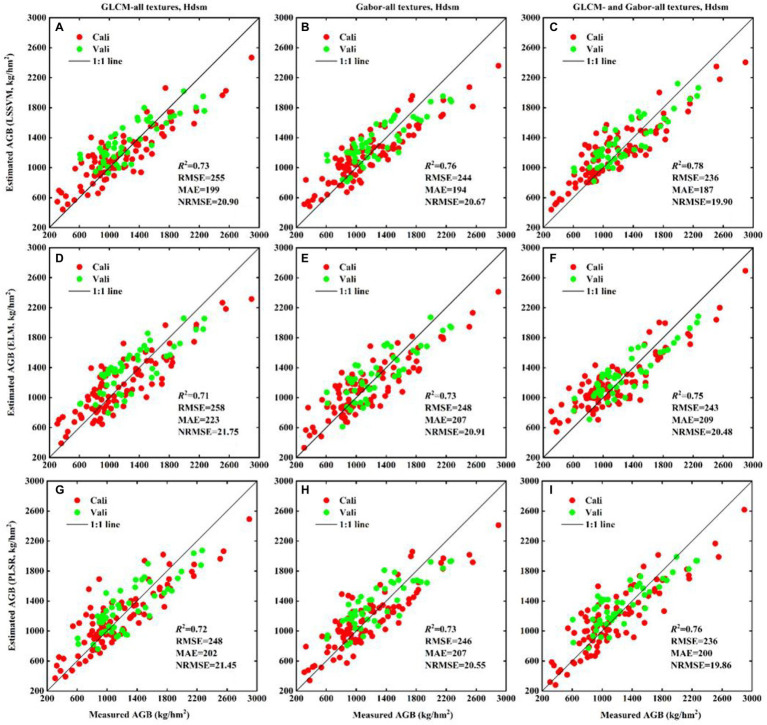
The fitted scatter plot of measuring and estimating potato AGB (kg/hm^2^) using different ground resolution GLCM-based textures, different scales Gabor-based textures and crop height. **(A–C)** LSSVM; **(D–F)** ELM; **(G–I)** PLSR. GLCM-all textures, Hdsm represent combination of different ground resolution GLCM-based textures and crop height. The estimation results of Cali and Vali are shown in Supplementary Table A3.

To determine whether the fusion of high-frequency information and vertical crop canopy structure information is helpful for AGB estimation, we estimate potato AGB by using (i) GLCM-based textures of different resolutions and crop height; (ii) Gabor-based textures of different scales and crop height; and (iii) GLCM- and Gabor-based textures of all forms and crop height. The results in Figure 11 show that (i) the LSSVM technique produces more accurate estimates of potato AGB (*R*^2^ = 0.73–0.78, RMSE = 236–255 kg/hm^2^, MAE = 187–199 kg/hm^2^, NRMSE = 19.90–20.90%); (ii) combining textures of different resolutions and scales separately with the crop height enhances the accuracy of AGB estimates (and more so for the latter); (iii) different textures combined with crop height produce the most accurate estimates of potato AGB for the same regression technique (*R*^2^ = 0.75–0.78, RMSE = 236–243 kg/hm^2^, MAE = 187–209 kg/hm^2^, NRMSE = 19.86–20.48%). More importantly, different textures combined with crop height are not underestimated when AGB exceeds 1,000 kg/hm^2^. The results in Figures 11B,E,H than those in Figures 11A,D,G, which validates the argument that multiscale Gabor-based textures are more promising than multi-resolution GLCM-based textures for estimating potato AGB.

## Discussion

The use of different varieties, planting density, nitrogen fertilizer, and potassium fertilizer leads to significant differences in the growth of potato crops. At present, to obtain AGB information, crop-growth monitoring is done mainly by optical remote sensing. However, this approach has limitations (Zhang et al., 2020) because optical VIs lose their sensitivity to AGB for high crop coverage, resulting in the underestimation of potato AGB in reproductive growth periods. To improve the AGB estimation of potato crops in multiple growth periods and investigate the feasibility of using digital cameras for AGB estimation, this study obtained RGB images of potato canopies from an economic UAV remote-sensing platform and directly extracted different texture features and crop height to estimate potato AGB.

### Response of crop height to AGB

Fluctuations in crop height can reflect crop health and nutritional status. At the same time, reasonable plant height is also the basis for stable and high-yield crops. Therefore, accurate acquisition of plant height information is vital for crop-growth monitoring and farmland production management (Lu et al., 2019). For example, Niu et al. (2019) and Yue et al. (2018a) used crop height to estimate maize and winter wheat AGB. Unfortunately, using crop height alone to estimate AGB during the whole growth period of maize and wheat may be of limited use because variations in height are not apparent in the later growth stage of maize and wheat, whereas AGB increases significantly (corn grain and ear formation). On the contrary, potato growth differs significantly from that of maize and wheat. In the early reproductive growth period, potato stem nodes elongate and leaves expand, increasing both AGB and crop height. However, in the later stage of reproductive growth, the underground tubers expand continuously so that the nutrients accumulated on the ground must be transferred underground, resulting in wilting and yellowing of the stems and leaves of above-ground potato plants. A small number of leaves even fall off, which reduces the crop height and AGB simultaneously, as shown in plot s03 of Supplementary Figure 1.

The visualization in Figure 5D shows that both crop height and AGB maintains a positive linear correlation (*p* < 0.01) in single or multiple growth periods, which is consistent with potato crop growth and indicates that potato crop height could be used for AGB estimation. The quantitative analysis results in Figure 11 show that the addition of crop height (*R*^2^ increases, RMSE and MAE decrease) in different models improves the accuracy of AGB estimation and reduces the underestimation of AGB, which also confirms the hypothesis that crop height may support AGB estimation in multiple growth periods of potatoes. Li et al. (2020b) reported that the potato crop height correlates significantly with the AGB of early maturing potato varieties (the potato plant and canopy branches grow synchronously), whereas the correlation with the AGB of late-maturing varieties decreases (when the potato plant grows to a certain height, the canopy is not well developed). However, the potato varieties Zhongshu 5 and Zhongshu 3 planted in this study are both early maturing varieties, and the conclusions obtained based on these varieties are consistent, namely, that a significant linear relationship exists between crop height and AGB (see Figure 5D). This shows that the difference in varieties is the key factor restricting the use of crop height to estimate AGB. Therefore, relying only on crop height to estimate the AGB of different varieties of the same crop seems ill-advised.

### Response of GLCM-based textures to AGB

The results show that the correlation between GLCM-based textures and AGB remains basically unaffected by the direction and window (Figure 6), which may be related to the principle of GLCM generation. It represents the occurrence frequency of a pixel pair. Little difference exists in the number of statistics in different directions and windows. Fu et al. (2021) and Yue et al. (2019) also reported that the correlation between GLCM-based textures and winter wheat AGB is largely unaffected by directions and windows. In contrast, the correlations between GLCM-based textures and AGB at different ground resolutions differ significantly (Figure 7), mainly because the amount of information contained in the potato canopy structure differs. Taking the G-band as an example, Table 3 shows the data range and variance statistics for each growth period. Although the range of G-band data obtained through the nearest pixel resampling method in each growth period remains basically unchanged, the variance decreases gradually with decreasing resolution, which is indicative of a decrease in the amount of information contained in the canopy image, modifying the correlations between GLCM-based textures and AGB at different resolutions.

**Table 3:** Statistical analysis of G band DN values at each growth period.

Images	P1		P2		P3	
	Data range	Variance	Data range	Variance	Data range	Variance
GDS1	255	66.92	255	60.72	255	53.74
GDS5	254	50.52	255	57.39	255	53.71
GDS10	254	50.61	243	57.34	250	43.32
GDS30	254	49.10	254	39.67	253	33.68
GDS60	252	48.54	241	37.64	252	32.75

We classified GLCM-based textures obtained with different ground resolutions into five categories: (i) GDS1, (ii) GDS5, (iii) GDS10, (iv) GDS20, GDS30, and (v) GDS40, GDS50, GDS60. As shown in Figure 7A, the GLCM-based textures of GDS1 remain strongly correlated with potato AGB because most pixels correspond to pure potato leaves or soil. However, the correlation between the GLCM-based textures of GDS5 and GDS10 and potato AGB begins to weaken and the sign of the correlation coefficient changes (Figures 7B,C) because a small number of pixels express potato canopy information in the original image. However, when using the nearest-neighbor resampling method, these pixels express soil information, so the mixed spectrum was extracted based on ROIs. Given that the potato row spacing was 60 cm, when the GDS changes from 20 to 30 cm, the fraction of soil pixels increases in the ROI (e.g., in the GDS30 cm image, the ROI may contain half vegetation and half soil, and the final extracted canopy spectra are similarly smoothed), which makes the correlation between GLCM-based textures of GDS30-cm and AGB greater than that between GDS20-cm and AGB (Figures 7D,E). However, when the ground resolution exceeds half the line spacing, a lower image resolution makes the fraction of soil in the pixels in the ROI more significant than vegetation, reducing the correlation between GLCM-based textures and AGB.

Consider the Dis of the B-band of plot s04 as an example. Under the same growth period, the Dis-texture gradually increases and then decreases from GDS1- to GDS30-cm. This shows that, when the GDS exceeds half the line spacing, the information contained in the ROI changes from high frequency (e.g., potato leaves) to low frequency (e.g., soil, shadow); that is, the texture changes from nonuniform (Supplementary Figure 2; GDS2-, GDS5-, GDS10-, GDS20-cm) to uniform (Supplementary Figure 2; GDS40-, GDS50-, GDS60-cm), which explains why the image resolution affects the correlation between GLCM-based texture and potato AGB. Potato AGB first increases and then decreases from the P1- to the P3-growth period, whereas the Dis-texture value of GDS1 (pure pixel; Supplementary Figure 2) maintains a significant positive correlation with AGB as the growth period changes (Figure 7A; Supplementary Figure 2; GDS1-cm). However, other ground resolution Dis-textures correlate negatively with AGB because most pixels in the ROI contain soil. Therefore, selecting an appropriate GDS is helpful for monitoring crop growth.

### Response of Gabor-based textures to AGB

The Gabor filter produces an effect very similar to the human visual response and is not sensitive to local illumination, making it highly suitable for extracting fine texture features. Following the work of others (Fu et al., 2020), we set four orientations and five scales to form a total of 20 filters (Figure 3). Based on these filters, we extract multiscale Gabor-based textures. The results show that the correlation between Gabor-based textures and potato AGB are significantly more affected by scale than by direction, which differs significantly from the results obtained from GLCM-based texture (Figures 8A–D). This was also confirmed by Fu et al. (2021).

Consider as an example the B-band of the n01 plot for the P1-P3 growth period. The results show that the amplitude images generated by the convolution of Gabor wavelet kernels of different scales with the RGB images have different spatial characteristics (Supplementary Figure 3). As mentioned earlier, potato AGB first increases and then decreases as the growth period advances, whereas B-S1-Con, B-S2-Ent, B-S4-Cor change with changing AGB, which means that the three correlate positively with AGB (Figures 8C; Supplementary Figure 2). Conversely, B-S3-Hom and B-S5-Sec maintain a negative correlation with AGB (Figures 8C; Supplementary Figure 2).

The above results show that extracting multiscale Gabor-based textures more finely describes variations in potato canopy structure. The results show that the sign of the correlation coefficients of the Ent-, Cor-, Sec-texture (GLCM-based) and AGB change (analogous to Gabor-based textures), which indicates that estimates of potato AGB made by using Gabor-based textures may differ from estimates of potato AGB made by using CLCM-based textures. Furthermore, the results shown in Figures 7E,F show that the multiscale Gabor-based texture is linear in both the potato single- and multi-growth period AGB. Unlike the results in Figures 6E,F, when the AGB exceeds 1,000 kg/hm^2^, the Gabor-based textures remain sensitive to AGB, which means that using multiscale Gabor-based textures to estimate AGB over multiple potato growth periods may produce more accurate result than GLCM-based textures, which is similar to the above hypothesis. The results in Supplementary Figure 3 show that the Gabor-based textures at different scales reflect the details of the potato canopy, which reminds us that the accuracy of Gabor-based textures must be evaluated at different scales to estimate potato AGB.

### Evaluation of accuracy of AGB estimation model

RGB images of the potato canopy typically consist of soil, stems, leaves, weeds, and shade (Supplementary Figure 1), and the fraction of each component changes with the advancement of the potato growth period. During the tuber formation period (P1), potatoes gradually close the ridge, and the shadow between the ridges appears clearly on RGB images. During the tuber growth period (P2), the branching of the potato canopy becomes maximal, as is the vegetation coverage, so the potato canopy almost covers the background soil. During the starch-storage period (P3), the distribution and transfer of assimilates on the ground causes the leaves to yellow and fall off, and the soil and weeds become apparent. These changes in the potato canopy can be captured by image texture features. In addition, given different varieties, planting densities, and fertilization treatments, significant differences appear in the potato canopy, even in the same growth period. Therefore, describing this change based solely on single-scale textures would be difficult. Figures 9, 10 show that using only a single type of texture to estimate AGB with different regression techniques is less effective than using multi-type textures (multi-resolution GLCM-based and multiscale Gabor-based textures).

The use of GLCM-based textures of GDS1 produces the most accurate AGB estimates when the ground resolution ranges from 1 to 5 cm. While the ground resolution ranges from 10 to 60 cm, the use of GLCM-based textures of GDS30 produces the most accurate AGB estimate (Figure 9; Supplementary A1). This differs somewhat from the results of Yue et al. (2019), who report that the textures GDS1 and GDS30 produce the most accurate AGB estimates of winter wheat. This discrepancy may be related to the difference in crop canopy structure and image down-sampling. The width of potato leaves (greater than or equal to about 5 cm for vigorous growth) far exceeds that of winter wheat leaves, which allows fine GLCM-based textures to be extracted from GDS5-cm images. In the present study, the information on the potato canopy structure obtained by the nearest pixel resampling method (the ROI spectrum is smoothed only at low resolution, such as less than 30 cm) differs significantly from the information on the winter wheat canopy structure obtained by Yue et al. (2019) through the average method (the degree of smoothness differs at different resolutions, and the smoothness increases as the image resolution decreases). Therefore, the estimation of potato AGB based on GDS30 cm images is inferior to that based on GDS5 cm images. Combining different ground resolution textures to estimate AGB is more accurate, so we conclude that textures with different GDS could provide complementary information from different perspectives to estimate AGB.

Similarly, we estimate potato AGB using single-scale Gabor-based textures (Figure 10; Supplementary A2). The results show that the estimation accuracy deteriorates with increasing scale (Figure 10; Supplementary A2), but samples with AGB exceeding 1,000 kg/hm^2^ are less underestimated, which is consistent with the results of Fu et al. (2021). As shown in Figure 3, an increased scale would make the Gabor convolution kernel larger, and the final Gabor-based textures are like the textures after down-sampling. As shown earlier, GLCM-based textures down-sampled from GDS1 to GDS5 and used to estimate AGB produce less accurate results. Fortunately, combining Gabor-based textures with different scales leads to more accurate AGB estimates than combining GLCM-based textures with different ground resolutions, which confirms the assumption of Section 3.3. The Gabor filter can be seen as a microscope sensitive to orientation and scale (it has good spatial resolution and direction selectivity); it can capture local structural information corresponding to spatial frequency and is robust against illumination and pose. These advantages show that Gabor filtering is a powerful tool to describe the local gray distribution of the image (i.e., variations in texture). Therefore, Gabor filtering at different scales could be used to extract finer textures from images.

We estimated AGB using the LSSVM, ELM, and PLSR techniques by combining all GLCM-based textures, all Gabor-based textures, and the combined textures with crop height (Figure 11; Supplementary A3). The results show that all models combined with crop height significantly improve the estimation accuracy, which confirms the importance of crop height for estimating potato AGB and confirmed the speculation in the introduction (Figure 5) that high-frequency information combined with vertical structure information may improve AGB estimates. Note that different textures combined with crop height produce the most accurate AGB estimates, and the samples are closer to a 1:1 line (Figure 11). Taking the 1,000 kg/hm^2^ AGB sample as the splitting point, we counted the NRMSE of the different models (Supplementary Figure 4). Using high AGB (1000–3,000 kg/hm^2^) samples as benchmarks, the accuracy of the AGB estimation model improves (i) by 22.97% (NRMSE) when using different types of textures combined with crop height (GLCM-all and Gabor-all, Hdsm) with the LSSVM technique as opposed to using only GLCM-all textures, (ii) by 14.63% compared with using only Gabor-all textures, (iii) by 9.74% compared with GLCM-all textures combined with crop height (GLCM-all, Hdsm), and (iv) by 8.18% compared with Gabor-all textures combined with crop height (Gabor-all, Hdsm). This study used one-year data to estimate potato AGB based on crop morphological characteristics accurately, but it lacked validation analysis of multi-year experiments. In future research, we will collect potato data from different places and years to evaluate the model’s performance, enhancing the reliability of the research results.

## Conclusion

This work evaluates the performance of GLCM-based textures of differing resolutions and Gabor-based textures of differing scales and their combination with crop height for estimating potato AGB. The results lead to the following conclusions:

1. The correlation between GLCM-based textures and AGB is unaffected by the direction and window, but it is more affected by the ground resolution. The correlation between Gabor-based textures and potato AGB is significantly more affected by scale than by direction.
2. The GLCM-based textures of GDS1and GDS30 produce the most accurate AGB estimates when the ground resolution ranges from 1 to 5 cm and 10 to 60 cm, respectively. However, the accuracy of potato AGB estimates based on Gabor-based textures gradually deteriorates upon increasing the convolution kernel scale.
3. Both multi-resolution GLCM-based textures and multiscale Gabor-based textures are better than single-type textures for estimating AGB, and multiscale Gabor-based textures are the best for estimating AGB.
4. Gabor-based textures combined with crop height produce more accurate AGB estimates than GLCM-based textures. Optimistically, combining two different types of textures with crop height solves the problem whereby potato samples with high AGB are underestimated, which is especially important for monitoring crop growth and advancing the development of precision agriculture.

## Data availability statement

The original contributions presented in the study are included in the article/Supplementary material, further inquiries can be directed to the corresponding author.

## Author contributions

HF, ZL, and GY designed the experiments. YL and HF collected the AGB, plant height and UAV digital images, analyzed the data, and wrote the manuscript. JY, XJ, and ZL provided comments and suggestions for the manuscript and checked the writing. All authors contributed to the article and approved the submitted version.

## Funding

This study was supported by the Key scientific and technological projects of Heilongjiang province (2021ZXJ05A05), the Key Field Research and Development Program of Guangdong Province (2019B020216001) and the National Natural Science Foundation of China (41601346).

## Conflict of interest

The authors declare that the research was conducted in the absence of any commercial or financial relationships that could be construed as a potential conflict of interest.

## Publisher’s note

All claims expressed in this article are solely those of the authors and do not necessarily represent those of their affiliated organizations, or those of the publisher, the editors and the reviewers. Any product that may be evaluated in this article, or claim that may be made by its manufacturer, is not guaranteed or endorsed by the publisher.
