# Supplementary material for: Estimation of potato above-ground biomass based on unmanned aerial vehicle red-green-blue images with different texture features and crop height
Source: Front Plant Sci. 2022 Aug 25;13:938216. doi: 10.3389/fpls.2022.938216 (PMC9452666; doi:10.3389/fpls.2022.938216)
Supplement: Supplementary file 1 [file Data_Sheet_1.docx]

Appendix

**Table A1.** Potato AGB (kg/hm^2^) estimates using selected GLCM-based textures.

| Data type | Data Set | LSSVM | | | ELM | | | PLSR | | | |
| --- | --- | --- | --- | --- | --- | --- | --- | --- | --- | --- | --- |
|  |  | *R*^2^ | RMSE | MAE | *R*^2^ | RMSE | MAE | *R*^2^ | RMSE | MAE |  |
| GDS1 | Cali | 0.69 | 280 | 213 | 0.68 | 280 | 230 | 0.64 | 299 | 241 |  |
|  | Vali | **0.61** | **257** | **222** | **0.62** | 282 | 245 | **0.60** | 271 | **226** |  |
| GDS5 | Cali | 0.68 | 284 | 226 | 0.62 | 305 | 245 | 0.63 | 306 | 248 |  |
|  | Vali | 0.59 | 260 | **222** | 0.54 | 314 | 257 | 0.58 | 267 | 232 |  |
| GDS10 | Cali | 0.64 | 302 | 249 | 0.59 | 322 | 257 | 0.61 | 317 | 282 |  |
|  | Vali | 0.56 | 283 | **227** | 0.51 | 338 | 266 | 0.55 | 276 | **228** |  |
| GDS30 | Cali | 0.64 | 303 | 263 | 0.60 | 318 | 267 | 0.62 | 307 | 270 |  |
|  | Vali | **0.61** | **258** | **211** | 0.59 | 276 | **225** | **0.60** | 261 | **227** |  |
| GDS60 | Cali | 0.63 | 307 | 268 | 0.59 | 327 | 275 | 0.60 | 312 | 259 |  |
|  | Vali | 0.54 | 292 | 247 | 0.47 | 296 | 254 | 0.53 | 282 | 249 |  |
| All | Cali | 0.72 | 268 | 225 | 0.69 | 227 | 223 | 0.70 | 270 | 216 |  |
|  | Vali | **0.66** | **246** | **205** | **0.62** | **253** | **204** | **0.64** | **250** | **209** |  |

**Note:** In Vali dataset, *R*^2^>0.60, RMSE<260 and MAE<230 are bolded.

**Table A2.** Potato AGB (kg/hm^2^) estimates using selected Gabor-based textures.

| Data type | Data Set | LSSVM | | | ELM | | | PLSR | | | |
| --- | --- | --- | --- | --- | --- | --- | --- | --- | --- | --- | --- |
|  |  | *R*^2^ | RMSE | MAE | *R*^2^ | RMSE | MAE | *R*^2^ | RMSE | MAE |  |
| S1 | Cali | 0.70 | 286 | 238 | 0.66 | 321 | 258 | 0.68 | 300 | 283 |  |
|  | Vali | **0.64** | **248** | **214** | 0.61 | 278 | 218 | 0.63 | 254 | 226 |  |
| S2 | Cali | 0.67 | 302 | 260 | 0.64 | 315 | 279 | 0.65 | 304 | 264 |  |
|  | Vali | 0.63 | 256 | 221 | 0.59 | 266 | 237 | 0.62 | 257 | 224 |  |
| S3 | Cali | 0.65 | 313 | 270 | 0.63 | 328 | 279 | 0.64 | 322 | 270 |  |
|  | Vali | 0.63 | 253 | 216 | 0.59 | 278 | 232 | 0.62 | 264 | 222 |  |
| S4 | Cali | 0.63 | 327 | 272 | 0.59 | 349 | 285 | 0.61 | 341 | 273 |  |
|  | Vali | 0.62 | 275 | 219 | 0.55 | 280 | 229 | 0.58 | 276 | 226 |  |
| S5 | Cali | 0.63 | 310 | 275 | 0.57 | 341 | 292 | 0.60 | 329 | 283 |  |
|  | Vali | 0.58 | 277 | 232 | 0.55 | 297 | 245 | 0.59 | 284 | 234 |  |
| All | Cali | 0.75 | 251 | 211 | 0.71 | 283 | 244 | 0.73 | 263 | 212 |  |
|  | Vali | **0.68** | **230** | **199** | **0.65** | **244** | **206** | **0.66** | **241** | **204** |  |

**Note:** In Vali dataset, *R*^2^>0.64, RMSE<250 and MAE<220 are bolded.

**Table A3.** Potato AGB (kg/hm^2^) estimates using textures and crop height.

| Data type | Data Set | LSSVM | | | ELM | | | PLSR | | |
| --- | --- | --- | --- | --- | --- | --- | --- | --- | --- | --- |
|  |  | *R*^2^ | RMSE | MAE | *R*^2^ | RMSE | MAE | *R*^2^ | RMSE | MAE |
| GLCM, Hdsm | Cali | 0.75 | 259 | 197 | 0.73 | 259 | 214 | 0.74 | 254 | 198 |
|  | Vali | 0.69 | 246 | 205 | 0.65 | 257 | 243 | 0.67 | 235 | 211 |
| Gabor, Hdsm | Cali | 0.77 | 249 | 233 | 0.74 | 257 | 212 | 0.75 | 250 | 207 |
|  | Vali | **0.72** | **204** | 203 | 0.68 | 230 | **197** | **0.70** | 236 | 208 |
| All, Hdsm | Cali | 0.78 | 244 | 202 | 0.76 | 253 | 216 | 0.76 | 238 | 211 |
|  | Vali | **0.74** | **219** | **195** | **0.70** | 222 | **194** | **0.72** | 233 | 206 |

**Note:** In Vali dataset, *R*^2^>0.70, RMSE<220 and MAE<200 are bolded.


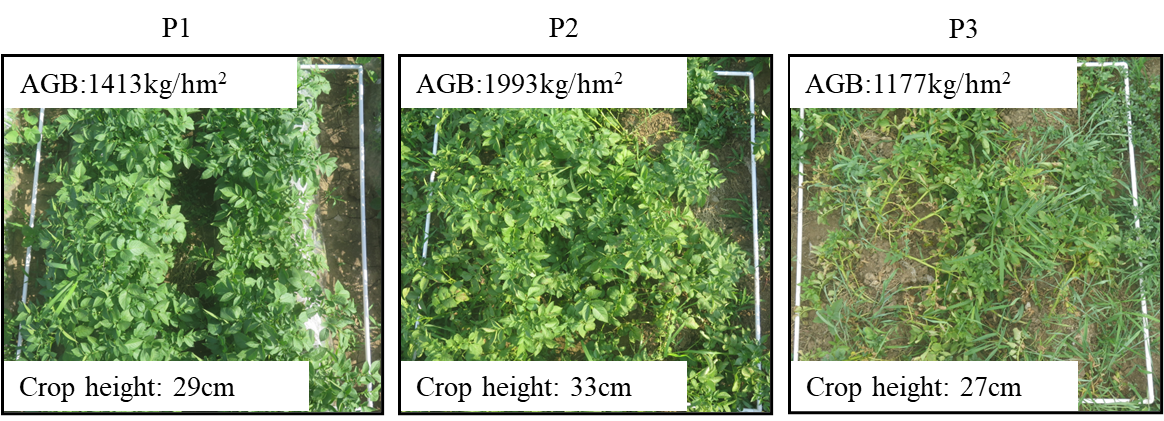


**Figure 1.** Potato AGB and crop height in s03 plot.


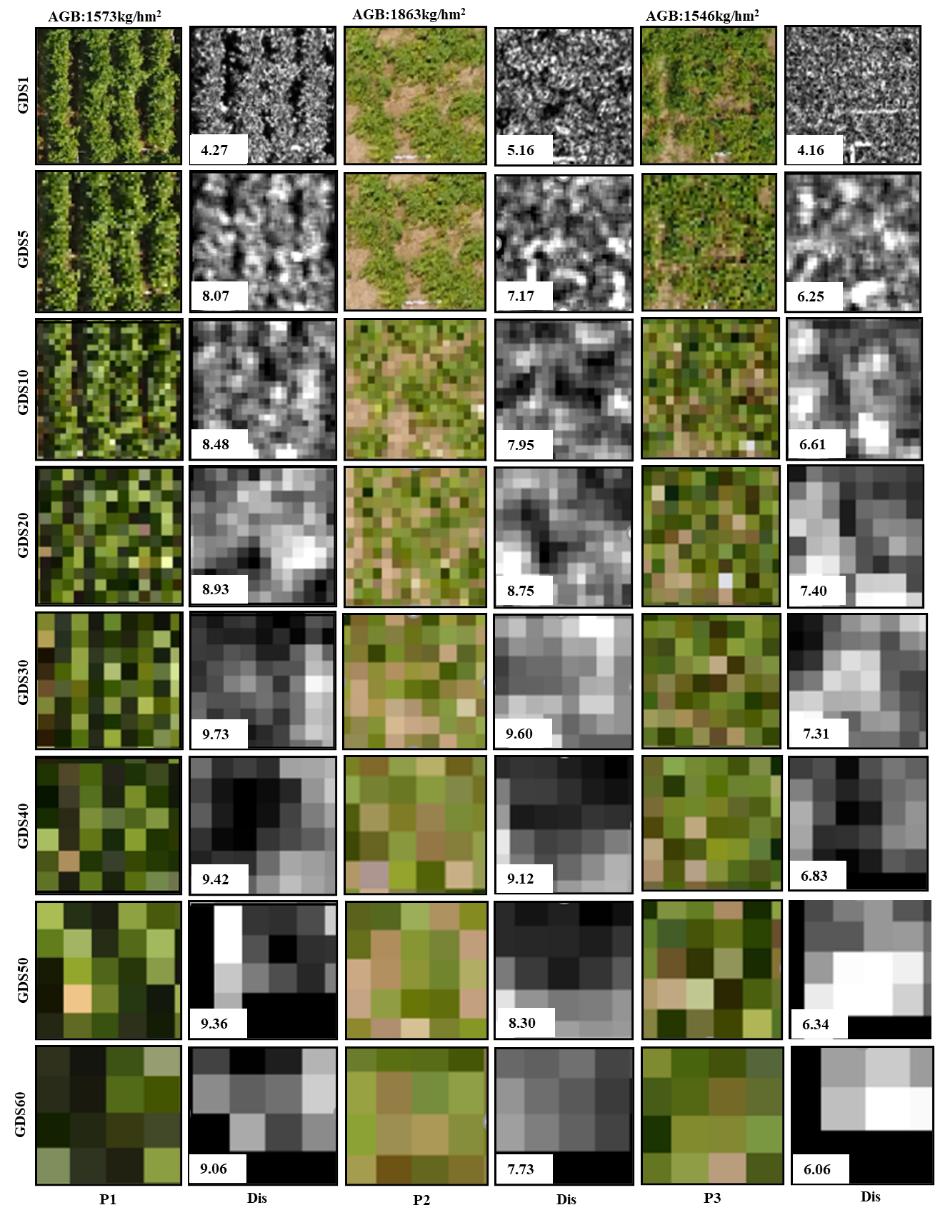


**Figure 2.** The RGB images and the Dis-texture of the B-band in the s04 plot.


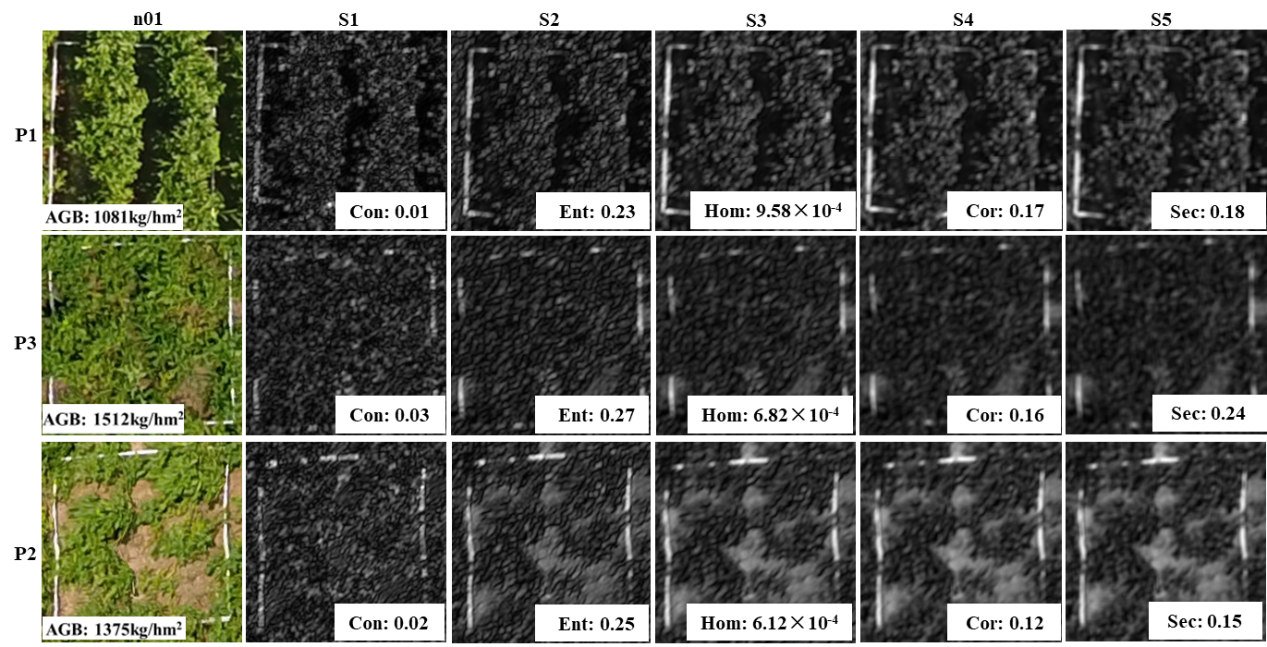


**Figure 3.** Gabor amplitude images and corresponding texture values at the P1-P3 growth period.


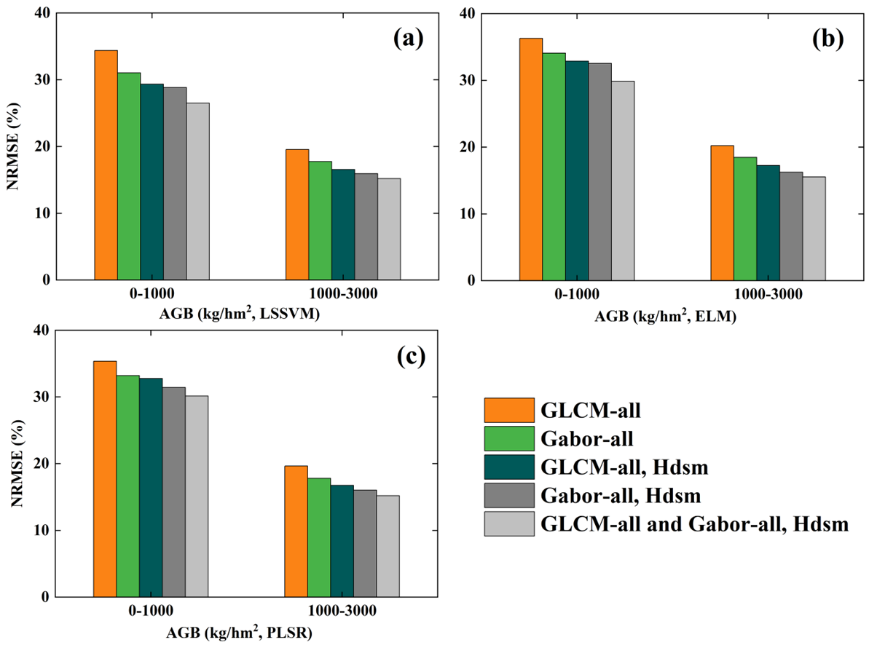


**Figure 4.** Descriptive statistics of regression accuracy (NRMSE) from the LSSVM, ELM and PLSR based on all GLCM-based textures (GLCM-all), all Gabor-based textures (Gabor-all), all GLCM-based textures combined with crop height (GLCM-all, Hdsm), all Gabor-based textures combined with crop height (Gabor-all, Hdsm) and different types of textures combined with crop height (GLCM-all and Gabor-all, Hdsm), respectively.
